# Anemia of Chronic Diseases: Wider Diagnostics—Better Treatment?

**DOI:** 10.3390/nu12061784

**Published:** 2020-06-16

**Authors:** Michał Wiciński, Grzegorz Liczner, Karol Cadelski, Tadeusz Kołnierzak, Magdalena Nowaczewska, Bartosz Malinowski

**Affiliations:** 1 Department of Pharmacology and Therapeutics, Faculty of Medicine, Collegium Medicum in Bydgoszcz, Nicolaus Copernicus University, M. Curie 9, 85-090 Bydgoszcz, Poland; wicinski4@wp.pl (M.W.); licznergrzegorz@gmail.com (G.L.); karol.cadelski@gmail.com (K.C.); tadekpiotr@wp.pl (T.K.); 2 Department of Otolaryngology, Head and Neck Surgery, and Laryngological Oncology, Collegium Medicum in Bydgoszcz, Nicolaus Copernicus University, M. Curie 9, 85-090 Bydgoszcz, Poland; m.nowaczewska@cm.umk.pl

**Keywords:** iron homeostasis, anemia, iron supplementation, oxidative stress, nutrition, hematological parameters, biochemical parameters, erythropoiesis

## Abstract

Anemia of chronic diseases is a condition that accompanies a specific underlying disease, in which there is a decrease in hemoglobin, hematocrit and erythrocyte counts due to a complex process, usually initiated by cellular immunity mechanisms and pro-inflammatory cytokines and hepcidin. This is the second most common type of anemia after iron deficiency anemia in the world. Its severity generally correlates with the severity of the underlying disease. This disease most often coexists with chronic inflammation, autoimmune diseases, cancer, and kidney failure. Before starting treatment, one should undertake in-depth diagnostics, which includes not only assessment of complete blood count and biochemical parameters, but also severity of the underlying disease. The differential diagnosis of anemia of chronic diseases is primarily based on the exclusion of other types of anemia, in particular iron deficiency. The main features of anemia of chronic diseases include mild to moderate lowering of hemoglobin level, decreased percentage of reticulocyte count, low iron and transferrin concentration, but increased ferritin. Due to the increasingly better knowledge of the pathomechanism of chronic diseases and cancer biology, the diagnosis of this anemia is constantly expanding with new biochemical indicators. These include: the concentration of other hematopoietic factors (folic acid, vitamin B_12_), hepcidin, creatinine and erythropoietin. The basic form of treatment of anemia of chronic diseases remains supplementation with iron, folic acid and vitamin B_12_ as well as a diet rich in the above-mentioned hematopoietic factors. The route of administration (oral, intramuscular or intravenous) requires careful consideration of the benefits and possible side effects, and assessment of the patient’s clinical status. New methods of treating both the underlying disease and anemia are raising hopes. The novel methods are associated not only with supplementing deficiencies, but also with the administration of drugs molecularly targeted to specific proteins or receptors involved in the development of anemia of chronic diseases.

## 1. Introduction

Erythropoiesis is a multi-stage process of multiplication and erythrocyte differentiation from hematopoietic stem cells, which normally takes place in the bone marrow of flat bones and the epiphyses of human long bones. A unique feature of stem cells is their ability to self-renewal and differentiation. From the hematopoietic stem cell, a myelopoietic stem cell is formed, which subsequently undergoes a transformation to the erythropoietic progenitor cell. It matures through successive divisions and becomes a precursor cell, demonstrating at this stage some characteristics of the final cell. Further maturation occurs through changing the nature of the cell nucleus from basophilic to acidophilic, up to its loss in order to minimize metabolism and inhibit the possibility of division. Mature, enucleated erythrocytes are released into the blood through the selective bone marrow barrier, formed by endothelial cells of the marrow vessels. Under pathological conditions, erythropoiesis can occur in the liver and spleen. Consequently, immature forms of erythrocytes appear in the peripheral blood, including reticulocytes and erythroblasts containing the cell nucleus. Erythropoiesis is subject to both local and systemic regulation. Although erythrocyte maturation is tightly programmed in the genome of hematopoietic stem cells, there are a number of factors that modify the process. These include adhesion molecules, cytokines, ligands and receptors binding them, tyrosine kinases activating transcription factors in the cell nucleus. Adhesive molecules are responsible for the adhesion of blood cells to the medium, while hematopoietic cytokines determine their survival and multiplication. Normal cells require constant cytokine stimulation, since the lack of such signal causes direction of the cell to the apoptosis pathway. Proper cytokine supply is the basic mechanism that regulates cell homeostasis and ensures stability in the structure and number of specific blood cells at a given site. The factor that regulates erythropoiesis at the systemic level is glycoprotein peptide hormone secreted by the liver (20%) and, to a greater extent, by type I peritubular cells of the interstitial tissue of the kidney cortex (80%), called erythropoietin [1].

It stimulates various stages of erythropoiesis due to binding to transmembrane EPO-R receptors, present mainly on precursor cells of the erythropoietic lineal, i.e., proerythroblasts. After ligand attachment, it creates a homodimer receptor and then activates tyrosine kinases JAK (Janus-activated kinase) and other transcription factors. It is noted that the amount of erythropoietin receptors is inversely proportional to the degree of erythrocyte maturity. They are no longer found in the cell membrane of reticulocytes and erythrocytes [1,2]. Conversely, the expression of this receptor in neoplastic cells appears to be a disturbing phenomenon. This hampers the administration of recombinant erythropoietin in patients with malignant neoplasm, which in this situation can promote tumor cell growth [3]. The situation triggering the release of erythropoietin is hypoxia of tissues of various origins (heart and lung diseases, smoking or being at high altitudes).

Therefore, EPO-R receptors occurs in tissues with high metabolism and high sensitivity to hypoxia, i.e., brain, heart muscle, skeletal muscle and kidneys [2]. In turn, the secretion of erythropoietin is disturbed in chronic kidney disease, where the production of this hormone is gradually reduced. Deficiency of erythropoietin or a lack of sensitivity to its target tissue is one of the development mechanisms of chronic diseases anemia. In addition to erythropoietin, other hematopoietic factors are necessary for the proper conduct of the erythropoiesis process. These include iron, vitamin B_12_, folic acid, vitamin C, vitamin B_6_, proteins and hormones. The absence of any hematopoietic factor or the appearance of factors directly damaging the bone marrow or mature erythrocytes will also result in the occurrence of anemia.

## 2. Iron Metabolism—General Remarks

Iron is one of the most important microelements of the body necessary for the synthesis of hemoglobin. In addition to its main building function, it also has a regulatory role. As a component of heme and cytochrome enzymes, it enables cellular respiration through electrons transfer in the electron transport chain, supports antioxidative processes and DNA synthesis (RNA reductase is an iron-dependent enzyme needed for DNA replication). The iron content in the human body oscillates around 3–4 g, of which approx. 2.5 g (65%) is in hemoglobin, approx. 400 mg (10%) in myoglobin, catalase and cytochrome, from 3 to 7 mg (0.1%) is bound by transferrin, while the rest is backup (25%). The stored iron can be divided into active (ferritin) and inactive (hemosiderin) pools [4].

The daily diet provides an average of 10–15 mg of iron, of which only 5–10% (i.e., 1 mg) is absorbed into the body. The human body uses 20–25 mg of iron daily for hemoglobin synthesis. Most of this element comes from the natural degradation of erythrocytes due to their damage or aging. However, there are situations in which its excessive accumulation in the body occurs. It may result from the excessive absorption of this element from the gastrointestinal tract (hemochromatosis), its excessive supply with food or from improper iron metabolism (shortage for erythropoiesis or excessive release from the liver or red blood cells). Currently, the ability of free iron to initiate oxidative stress is widely discussed, consisting mainly of the production of free radicals, including reactive oxygen species, which damage nucleic acids, lipids and proteins contributing to carcinogenesis [5,6].

## 3. Iron Absorption and Characterization of Proteins Involved in the Metabolism

Absorption of iron in the gastrointestinal tract is a complicated process involving many proteins (DMT1 protein, ferroportin, ferritin, hepcidin, hephaestin, transferrin, lactoferrin). At the cellular level, deficiency of this element increases the synthesis of the transferrin receptor and increases the absorption of iron in the gastrointestinal tract. While at the systemic level, its deficiency significantly reduces the synthesis of ferritin (unless the deficiency is accompanied by inflammation that is present at the same time, because ferritin is an acute phase protein). The so-called active iron, i.e., rapidly exchanging, is associated with ferritin and is found primarily in hepatocytes and liver macrophages, spleen, bone marrow and muscles. The role of ferritin is primarily intracellular iron storage, which prevents the formation of reactive oxygen species (ROS) generated by the Fenton reaction. The ferritin molecule consists of soluble protein—apoferritin and the internal part containing iron ions. The protein part of ferritin is constructed from 24 chains consisting of light L and heavy H subunits forming a quadruple helix. The H subunit has oxidoreductive properties, which are attributed to ferroxidase center. Fe^2+^ ions after penetrating through the apoferritin coating into ferroxidase centers are oxidized to Fe^3+^ trivalent form. Inflammation increases the expression of the H subunit of ferritin, which, thanks to ferroxidase centers, has the ability to sequestrate iron and protect against oxidative stress. The consequence of this phenomenon is a deficit of available iron and anemia observed in chronic inflammation. Under physiological conditions, only the glycosylated form of the L ferritin subunit is present. The quantitative proportions of light and heavy chains forming ferritin differ depending on the state of iron homeostasis and the type of tissue. The H subunit predominates in cells requiring high iron availability, such as erythrocytes and myocardial cells. In iron storage organs (liver, spleen), ferritin is mainly built of the L subunit, which does not contain ferroxidase centers, and its main role is to store bioavailable iron atoms [7,8]. The role of ferritin as a carrier protein has recently been confirmed. It is assumed that ferritin can transport 100 to 1000 times more iron than transferrin. To serve as an iron-providing protein, ferritin must be released from the cells in which it is produced. Some studies suggest that intracellular ferritin may be transported to the lysosome for its degradation and release of iron, which is then recycled. This process is called ferritinophagy. The iron exported to the cytosol is then used in several physiological processes that include the synthesis of mitochondrial heme and erythrocyte differentiation. In the transport of cytosolic ferritin to the lysosome, a nuclear receptor co-activator 4 (NCOA4) is required, which binds to ferritin through the C-terminal domain and delivers it to the forming autophagosome. NCOA4 activity and severity of ferritinophagy are regulated by intracellular iron levels. Under the conditions of full availability of this element, ubiquitin E3 ligase (HERC2) binds NCOA4 and leads to its proteasomal degradation. The reduced level of NCOA4 promotes stabilization of ferritin by reducing the level of intracellular iron and inhibiting ferritinophagy, which, by producing reactive oxygen species (ROS), causes ferroptosis. NCOA4 deficiency in animal models is manifested in the inability to degrade ferritin and leads to a decrease in the intracellular iron bioavailability. Ferritinophagy is involved in NCOA4-mediated erythropoiesis, which enables the release of iron from ferritin, which is necessary for the synthesis of mitochondrial heme. Therefore, NCOA4 deficiency may be one of the mechanisms responsible for the development of anemia of chronic diseases [9,10]. The iron in foods is present in the form of the Fe^3+^ ion, which under the influence of gastric juice and Fe reductase (DCYTB) is reduced to the Fe^2+^ ion [11]. Its absorption depends on the body’s demand for this element and takes place mostly in the proximal part of the small intestine, where optimal conditions prevail. Bile secreted by the liver enters the duodenum, where high-affinity bonds form between iron and bile salts. As a result, well-soluble iron compounds (cation-bile salt complexes) are formed, which reach the intestinal mucosa more easily. Bile, by binding iron ions, thus prevents the precipitation of sparingly soluble iron oxides (Fe_2_O_3_). Bile salts are therefore important buffers of iron ions, increasing their absorption in the digestive tract [12,13]. At the time of iron deficiency, the expression of DMT1 (divalent metal transporter 1) increases, which is responsible for its increased absorption. At the same time, the ferroportin-controlled release of iron into the blood increases. Hepcidin—a 25-amino acid peptide predominantly synthesized by liver cells that antagonizes ferroportin, plays a key role in regulating iron metabolism [14,15,16]. It binds to ferroportin, which limits the release of iron from enterocytes, monocytes, macrophages and hepatocytes into the blood. The amount of secreted hepcidin correlates with the intensity of erythropoiesis and iron reserves in the human body, and most importantly, its synthesis and release are stimulated by inflammatory cytokines, which are produced in excess in the course of chronic diseases [17]. The iron already released into the circulation is oxidized with the participation of hephaestin (HEPH) to the Fe^3+^ ion and combined with transport proteins, i.e., transferrin and lactoferrin. Transferrin is a glycoprotein synthesized in the liver by hepatocytes, glial cells, lymphocytes, Sertoli cells and mammary gland cells. The rate of synthesis of this protein, like hepcidin, is strictly conditioned by the body’s demand for iron and the concentration of this element in the blood serum. Under normal conditions, 30 to 40% of transferrin is saturated with iron, the remaining 60% is in the form of apotransferrin [18]. The mechanism that secures the loss of these proteins in the urine during glomerular filtration is their high molecular weight of about 80 kDa. The mature transferrin molecule is formed by a long 679 amino acid polypeptide chain, which is made up of two similar N- and C-terminal domains connected by a short peptide. Each of these domains, by adopting an alternating β and α structure stabilized by 19 disulfide bridges, has one hydrophilic iron ion binding site, which ultimately allows the transport of two iron atoms by one transferrin molecule [19]. The iron carriers reach the precursor cells of the erythropoietic line (proerytroblasts), which express on their surface transferrin receptors, thanks to which fusion with iron transporting proteins is possible. The iron released by endocytosis is used for hemoglobin production and partly stored by ferritin. The iron-depleted transferrin (apotransferin) leaves the precursor cell and goes back to the bloodstream, where it functions as described above [20,21]. The presence of transferrin receptors is mainly represented by tissues with high metabolism and cells that are actively dividing. This partly may explain the increase in iron demand in people suffering from certain types of neoplasm which results not only from the constant use of this element in the production of hemoglobin, but also DNA synthesis by malignant cells [18]. Despite the fact that in the case of malignant neoplasms this is not one of the main causes determining the occurrence of anemia of chronic diseases (more often caused by chemotherapy-induced myelosuppression, blood loss, compromised iron distribution, etc.), attempts are being made to use drugs targeting the transferrin receptor, which blockade would interfere with the metabolism of actively dividing cells [22]. Nevertheless, transferrin itself as a transporter protein can be used to bind oxidative stress-generating free iron or to transfer a molecularly targeted drug to malignant cells [23].

## 4. Regulation of Iron Metabolism at the Cellular and Systemic Level

The expression of key proteins involved in iron absorption is controlled at many levels: transcriptional, post-transcriptional and post-translational. It turns out that the individual stages of the iron absorption process are subject to separate regulations. In the last decade, particular attention has been paid to the effects of hypoxia and molecular mechanisms of iron homeostasis. The transcriptional regulation of genes involved in the absorption and transport of iron across the intestinal mucosa was largely unknown until the HIF-2 transcription factor was discovered. HIF transcription factors are central mediators of cellular adaptation to hypoxia. They form heterodimers containing a regulatory α subunit, responsive to iron deficiency and hypoxia, and a β subunit with constitutive expression known as the aryl hydrocarbon receptor nuclear translocator (ARNT). There are three α subunit isoforms (HIF-1α, HIF-2α and HIF-3α) that are regulated at post-translational level [24]. HIF-1 has been the most widely studied subunit to date. HIF-1 has been shown to be involved in angiogenesis, glycolytic metabolism, apoptosis and cellular stress [25]. HIF-1 has also been shown to regulate expression of transferrin 1 receptor (TfR1) and expression of heme oxygenase 1 (HO-1) [26,27]. HIF-2 plays a key role in adult erythropoiesis by increasing the hepatic production of erythropoietin and the absorption of iron from the gastrointestinal tract. In enterocyte, HIF-2 regulates iron absorption by directly activating transcription of the divalent metal transporter 1 (DMT1), iron reductase (DCTYB) and ferroportin (FPN). In addition, HIF-2 inhibits hepcidin production in the liver, which molecularly binds to the iron exporter, i.e., ferroportin [28]. In patients with chronic kidney disease (CKD), the lack of EPO production by the interstitial tissue of the kidneys results in defective erythropoiesis, which consequently leads to anemia. Under physiological conditions, exposure to hypoxia stabilizes HIF-2 in EPO-producing kidney cells, leading to increased production of this hormone. At normal oxygen partial pressure (21%), the α subunits are hydroxylated and quickly degraded by iron and oxygen dependent enzymes (prolyl 4-hydroxylase, PHD). When the iron level is low, the prolyl hydroxylase activity decreases, thus the α subunits are destroyed much more slowly. During hypoxia, the hydroxylation (stabilization) of the α subunit is inhibited, which enables its translocation to the nucleus and interaction (dimerization) with the β subunit. The functional heterodimer moves to the nucleus to regulate the transcription of target genes (DCYTB, DMT1, FPN) by binding to specific sequences called hypoxia-responsive elements (HRE). Direct binding of the HIF heterodimer to HRE elements in the promoter regulatory regions has been demonstrated for DCYTB and DMT1. Their induction probably mediates the increase in iron absorption observed in conditions with low iron and oxygen content [29]. The increase in the number of CKD cases underlines the need for novel therapeutic approaches for treating anemia. Since the discovery of HIF, a concept of HIF stabilization has been suggested to support EPO production in CKD anemia. This is now possible thanks to a new class of drugs—PHD inhibitors or HIF stabilizers that prevent degradation of the HIF α subunit via the proteasome. This can improve the quality of life of patients with CKD by avoiding multiple transfusions, iron supplementation, or reducing doses of erythropoiesis stimulating agents [30].

**Figure 1:**
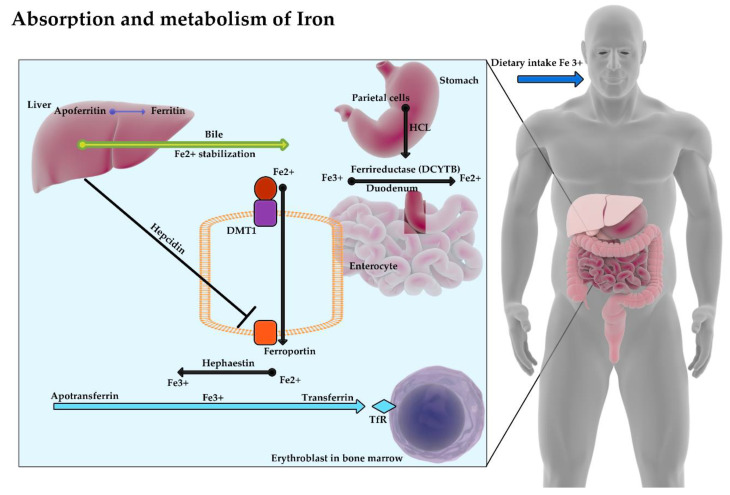
Fe^3+^ ions with food reach the stomach, where with the participation of hydrochloric acid and then (in duodenum) with ferric reductase enzyme (DCYTB) are transformed into Fe2+ ions. Bile secreted by the liver stabilizes iron by inhibiting its precipitation in the form of oxides. Thanks to DMT1 protein (divalent metal transporter 1), iron passes into the enterocyte and then it is released into the blood by ferroportin. Hepcidin produced by the liver limits the release of iron from enterocytes into the blood by binding to ferroportin. The divalent iron released into the blood is reoxidized with the contribution of hephaestin and ceruloplasmin to a trivalent ion, which, when combined with apotransferrin, forms transferrin and reaches the erythroblasts in the bone marrow.

The absorption and metabolism of iron have been presented in Figure 1.

## 5. Pathogenesis of Anemia of Chronic Disease

Anemia of chronic disease, also known as secondary anemia is the most common hematological disorder of the erythropoietic line after iron deficiency anemia in the world [31]. There is a permanent increase in the incidence of this type of anemia which is associated with the aging of the population and the tendency to develop chronic diseases, mainly malignant tumors and chronic kidney disease. Its incidence varies from 40% in patients with solid tumors and reaches almost 100% among patients with leukemia or lymphoma [32]. It was proven that anemia significantly worsens the quality of life of patients with chronic diseases, and in some types of cancer (lung cancer, locally advanced head and neck squamous cell carcinomas, cervical cancer) is an independent adverse prognostic factor [33,34,35]. It is currently assumed that there are several mechanisms leading to overt anemia of chronic diseases. They are interrelated. The first refers to the reduced iron reservoir needed for heme synthesis. This deficiency results from excessive production of hepcidina regulatory protein produced by the liver under the influence of cytokine stimulation, which inhibits the absorption of iron from the gastrointestinal tract and at the same time reduces its release into the blood [36]. Inflammatory cytokines, i.e., IL-1, IL-6, IL-10 and IFN-γ or TNF-α are the main factors contributing to the increase in hepcidin gene expression [37]. The second mechanism involved in the pathogenesis of anemia of chronic diseases is impaired production of erythropoietin. It results either from the advancement of chronic kidney disease being a consequence of the coexistence of other diseases (e.g., diabetes), whose natural course is associated with progressive nephropathy, or the direct action of the above-mentioned proinflammatory cytokines, which inhibit the expression of erythropoietin and, consequently, impede erythropoiesisin response to hypoxia [38,39]. In addition, the presence of proinflammatory cytokines reduces the sensitivity of proerythroblasts to erythropoietin and significantly reduces the survival of mature erythrocytes in peripheral blood [31]. The relationship between the stage of cancer and the concentration of endogenous erythropoietin and the degree of anemia associated with it is currently being intensively studied. Receptors for erythropoietin are located not only on the surface of erythrocyte precursors, but also on cells of certain types of tumors (breast cancer, prostate cancer, squamous cell carcinomas of the head and neck, multiple myeloma) and capillary wall cells of selected tumors [40,41,42]. Thus, recombinant analogues and derivatives of human erythropoietin used in the treatment of anemia of chronic diseases can promote tumor growth and immortality (including through pro-angiogenic and anti-apoptotic effects). Therefore, effective EPO-R blockade remains to be considered, which may in the future become one of the methods of cancer treatment.

## 6. Diagnosis of Anemia of Chronic Disease

The diagnosis of anemia of chronic diseases is associated with the exclusion of other types of anemia, including iron deficiency anemia. Although both types of these disorders share a deficiency of this element, there are several important features that allow them to differentiate. When examining the causes of anemia of chronic diseases, one should remember about disturbed hemoglobin production, the hemolytic and deficient component of this disease as well as the complicated humoral and cellular regulation of the hematopoietic process. The emergence of novel biochemical and molecular markers of secondary anemia is associated with a better understanding of the biology of cancer and the diseases it accompanies. Anemia of chronic diseases is described among people with infectious diseases of viral (e.g., HIV), fungal, parasitic and bacterial (e.g., tuberculosis) etiology [43,44]. People who undergo immunosuppressive therapy after organ transplantation or for the treatment of autoimmune diseases (rheumatoid arthritis, systemic lupus erythematosus, etc.) are also often affected by secondary anemia [45,46,47].

The Table 1 below presents the parameters differentiating iron deficiency anemia from anemia of chronic diseases, including the assessment of blood counts, concentration of selected hematopoietic factors and biochemical parameters as well as the presence of other chronic diseases.

## 7. Treatment of Anemia of Chronic Diseases

**Figure 2:**
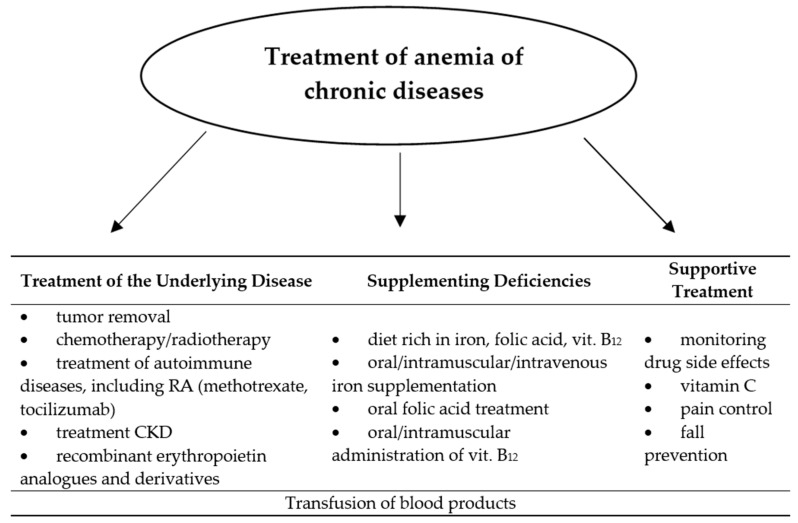
Treatment of anemia of chronic diseases.

Along with the diagnosis of secondary anemia, it is necessary to specify the stage and previous way of treatment of the underlying disease, which is associated with it, and the extent of deficiencies of basic hematopoietic factors. Although the exact management of this disease has not yet been defined, two directions of action to treat this type of anemia are still being adopted: silencing the underlying disease and supplementing deficiencies [48,49]. These actions can be taken in parallel or individually, depending on the severity of the anemia and the patient’s condition. Sometimes it is necessary to transfuse blood products to the patient, which is most often the case in advanced malignant disease. However, this gives a short-term effect and requires hospitalization [50]. The neoplastic process is associated with a high demand of rapidly dividing cells for hematopoietic factors, which become necessary not only for the synthesis of nucleic acids or proteins, but also the supply of the tumor with blood itself. The vicious circle mechanism occurs when the neoplastic cells begin to produce proinflammatory cytokines and other bone marrow-damaging substances or mature erythrocytes. With the growth of the tumor and the occurrence of distant metastases, the appearance of anemia becomes likely. Therefore, focus on causal treatment is the primary treatment for the anemia of chronic disease. Nevertheless, extensive iron (oral or intravenous), vitamin C, folic acid and vitamin B_12_ supplementation should be considered, and in advanced chronic kidney disease and in malignancy during chemotherapy, additionally administration of erythropoietin preparations [50,51,52]. In clinical observation, the implementation of such treatment significantly delays or prevents the occurrence of anemia of chronic diseases. Administration of ferrous chloride instead of ferrous sulfate or gluconate allows to bypass gastrointestinal side effects resulting from oral supplementation of this element [53]. Vitamin C has been shown to facilitate its absorption in the gastrointestinal tract, thanks to which it is often found as an additional component of iron preparations. In addition, vitamin C has an antioxidant effect. If oral iron therapy is insufficient, intravenous administration in the form of an iron hydroxide complex with sucrose or polyisomaltose should be considered [20]. Supplementation with vitamin B_12_ and folic acid is particularly justified during immunosuppressive therapy, including methotrexate in patients with rheumatoid arthritis. Perhaps the intensive development of immunotherapy in neoplastic and autoimmune diseases will allow treatment more specifically focused on the pathomechanism of anemia of chronic diseases. The use of an anti-IL-6 receptor (tocilizumab) antibody in anemia associated with rheumatoid arthritis, confirming its positive effect on hemoglobin increase [54,55]. There are also reports of the possibility of blocking the action and production of hepcidin, whose presence in the anemia of chronic diseases impairs the absorption and release of iron into the blood. Such management has been used successfully in mice given anti-hepcidin antibody [56]. At present, hemodialysis is the only possible way to eliminate hepcidin from blood serum in humans, which in this situation brings more complications than benefits for the patient [57]. The most elucidated method of treating anemia associated with neoplasm, in addition to the hematopoietic factors supplementation and transfusion of blood-derived preparations described above, is the use of recombinant erythropoietin analogues and derivatives. Currently, three preparations of human recombinant erythropoietin are available on the market: rhEPOα and rhEPOβ, which is a product of genetic engineering, and a modified derivative of human erythropoietin—darbepoetin, which has a much longer half-life and therefore requires less frequent administration than the other two forms [58,59]. Numerous clinical studies have proved the positive effect of these drugs on the increase of hemoglobin and improvement of well-being among patients receiving chemo- and radiation therapy. An indication for their use cannot, however, be prophylaxis of anemia or improvement of the quality of life [50]. Due to the presence of erythropoietin receptors on cells of certain types of malignant tumors (lung cancer, breast cancer, squamous cell carcinomas of the head and neck area), EPO preparations in these patients may, despite a temporary improvement in well-being, cause disease progression or premature death [40,41]. The latest guidelines limit the use of rhEPO preparations for the treatment of anemia associated with chronic kidney disease in persons without a history of neoplastic disease, and for the treatment of anemia associated with chemotherapy of solid tumors, but only among patients with hemoglobin in the range of 9–11 g/dL and only until reaching the concentration of 12 g/dL. Administration of erythropoietin derivatives requires the constant monitoring of blood counts, including the red cell system, and observation for possible long-term adverse effects of these drugs (deep vein thrombosis, stroke, myocardial infarction) [58]. The Figure 2 below shows methods of treating anemia of chronic disease.

## 8. The Role of Nutrition and Supplementation of Hematopoietic Factors in the Treatment of Anemia of Chronic Diseases

The correct diet pattern of an adult should take into account the real demand of the system for individual micronutrients, bioelements or vitamins. Thanks to a well-balanced and varied diet, maintaining stability of red blood cell parameters becomes much easier, even if there is periodic blood loss, impaired production or excessive destruction, which occurs in the anemia of chronic diseases. Although isolated deficiencies of hematopoietic factors in most situations determine the nature of anemia, in the case of anemia of chronic diseases are only part of the complex pathomechanism of this disorder. Among the causes of anemia of chronic diseases, a deficit of several hematopoietic factors is sooner perceived than isolated deficiency of each individual. This justifies extensive iron, folate and vitamin B_12_ supplementation during the treatment of this type of anemia [60,61].

## 9. Oral Iron

Iron offers the widest selection of administration routes. It is available in oral, intramuscular and intravenous forms. Each of these possible routes of administration of this element requires consideration of the potential benefits that the patient may receive, but also of the risks associated with side effects. Iron is usually supplemented by the oral route in the form of tablets, dragees or capsules, although its supplementation is associated with a relatively high risk of side effects (according to some reports, even in about 30% of patients) [62]. These mainly include gastrointestinal disorders (nausea, vomiting, constipation or diarrhea), the severity of which generally depends on the type of preparation used. Less common complaints are skin allergic reactions in the form of itching, rash or erythema. When iron is administered orally, black stools appear, which is associated with the presence of iron sulphides. The absence of such a color indicates then irregular use of the drug. The absorption of iron from the gastrointestinal tract in most people with anemia of chronic diseases is impaired, which is associated, among others with hepcidin production. Considering the other factors affecting its absorption (plant or animal origin of iron, taking drugs interacting with iron preparations), one can expect difficulties in obtaining effective supplementation, which, as expected, would not require another way to supplement the deficiency of this element. Although oral iron administration can be troublesome for the patient, the side effects are certainly not life threatening. This feature gives this form of supplementation an advantage over others, unless there are indications for intramuscular or intravenous supplementation of this element. Oral preparations are also safer because they decrease the presence of a free fraction of iron in the blood known as non-transferrin bound iron (NTBI). Free iron predisposes to the occurrence of infections, exacerbates the symptoms of sepsis and intensifies inflammatory reactions and oxidative stress, thus damaging the endothelium, which accelerates the process of atherogenesis. Macio and Madeddu evaluated dietary supplements taken by older people that would stimulate erythropoiesis and antioxidant activity, so that they could potentially be used to treat anemia of chronic diseases. Salidroside obtained from the extract of Rhodiolarosea, which stimulates the synthesis of erythrocytes, belongs to such substances. Cyanobacteria spirulin also raises high hopes. Its administration to humans increases the mean hemoglobin content of red blood cells [20].

## 10. The Impact of Diet and Drugs for the Treatment of Iron Deficiency

Iron consumed by humans occurs naturally in two forms (heme and non-heme). Heme iron is very well absorbed by the human body and is found in the largest amounts in red meat and offal such as the liver, kidneys, heart. Non-heme iron is, however, poorly absorbed and is found mainly in products of plant origin. Its bioavailability increases significantly when the plant also contains vitamin C. It has been shown that the administration of ascorbic acid at a dose of 250 mg significantly improves gastrointestinal absorption of iron administered orally. In addition, vitamin C facilitates its uptake in the bone marrow, thus accelerating erythropoiesis [63]. Plant-derived products with a high iron content include dry pulses, parsley, cocoa, nuts and spinach. Iron absorption is inhibited by drugs such as calcium and magnesium carbonate, pancreatic extracts, cholestyramine, neomycin, tetracyclines and proton pump inhibitors. The abovementioned drugs form hard-absorbed compounds or complexes with iron. Therefore, it is important to keep at least 2 h between taking these drugs and taking iron preparations. The same principle should also be adopted when consuming milk and its products, certain vegetables and drinking coffee or tea, which contain substances perfectly complexing iron, i.e., phytates, phosphates and oxalates. Taking oral iron preparations in the form of an iron hydroxide III complex with polymaltose together with food or medicines (tetracyclines, antacids) is acceptable and does not expose the patient to the risk of interaction. Iron alone, in turn, can reduce the absorption of simultaneously administered zinc salts, tetracyclines, bisphosphonates, entacapone, methyldopa, carbidopa, levodopa, fluoroquinolones, penicillins, penicillamines and thyroxine, which also requires a 2-h interval between their administration and iron intake. Adverse interaction is taking iron together with nonsteroidal anti-inflammatory drugs. The risk of irritation of the gastrointestinal mucosa increases then, which may promote the development of peptic ulcer disease and secondary anemia. The presence of peptic ulcer disease or other gastrointestinal diseases accompanied by inflammation (Crohn’s disease, ulcerative colitis) disqualify the patient from oral iron supplementation, as this increases the risk of bleeding. High bioavailability of iron adversely affect the course of diseases associated with chronic inflammation. Iron sequestration taken by host cells is a strategy that prevents the proliferation of pathogens. It has long been known that the development of bacteria depends on the presence of iron, and its elimination effectively limits the growth of pathogens. It appears that iron chelating compounds play a key role in this process. Well-known polyphenols have long been recognized as antimicrobials. The iron-polyphenol complex cannot be absorbed by epithelial cells and is excreted in faeces, suggesting that intestinal bacteria are also unable to absorb iron chelated by polyphenols [64]. During studies using mouse models of colitis, it was found that increased oxidative stress caused by oral iron administration is a major cause of exacerbation of the disease. Changes in the intestinal microflora, consisting in the uncontrolled growth of pathogenic strains, largely contributed to the exacerbation of inflammation in the large intestine in mice. The intestinal microflora absorbs the iron needed for growth thanks to low molecular weight chelators, called siderophores. The most common siderophore is enterobactin, which is found mainly in gram-negative bacteria such as Salomenlla typhimurium. In inflammatory bowel disease, lipocalin-2 is secreted by epithelial cells. It binds enterobactin and reduces the availability of iron for the intestinal microflora. The production of lipocalin-2 is an innate defense system used to limit the growth of microorganisms. However, there are pathogens that have developed modified siderophores which are insensitive to lipocalin-2. These include Salmonella enterica, which produces salmochelin instead of enterobactin. Pathogens that are not dependent on enterobactin then gain an advantage due to commensal growth [65]. Heme is an important source of iron for both the host and intestinal microorganisms. It has been proven that pathogenic strains grow particularly well in places rich in heme. Constante et al. showed that a diet rich in heme in mice with sodium sulfate-induced colitis (DSS) changes the composition of the colonic microflora by increasing the number of Proteobacteria, eliminating most of the positive G bacteria from the Firmicutes group, and increasing the protective effect of probiotics [66]. In inflammatory bowel disease, the role of the intestinal microflora should be appreciated and the possibility of using natural iron chelating agents to suppress the inflammatory response of immune cells while inhibiting the growth of pathogenic pathogens should be considered [67].

## 11. Parenteral Iron

If anemia is significant enough that it requires rapid correction of iron deficiency, its supplementation becomes necessary by the intramuscular or intravenous route. According to recommendations parenteral iron administration is only permissible in certain situations (in the case of intolerance to oral preparations, their impaired absorption from the gastrointestinal tract or the current high demand for this element, especially in patients with blood loss or treated with preparations stimulating erythropoiesis) [68]. The undisputed advantage of parenteral iron deficiency supplementation is the administration and dosage regimen. While oral treatment must be regular and long-term, intramuscular or intravenous therapy is generally interventional in nature, consisting of higher doses of iron being administered once or several times. In a randomized clinical trial, Quinibi et al. demonstrated the superiority of the iron III hydroxide and carboxymaltose complex applied intravenously over oral preparations, thus obtaining higher hemoglobin and ferritin levels while reducing adverse effects on the patient [69]. Similar conclusions were drawn from the Macdougall review, where better tolerance of new intravenous iron preparations such as carboxymaltose or ferumoxytol was demonstrated. This is probably due to the fact that the above preparations do not cause anaphylactic reactions and do not require the administration of a test dose [70]. In fact, however, parenteral iron administration has more serious side effects that are not seen in case of oral administration. Therefore, intravenous administration of iron preparations requires the greatest caution, as apart from the occurrence of hypersensitivity reactions, a sharp increase or decrease in blood pressure may occur. Moreover, the parenteral administration of iron reduces its absorption from the gastrointestinal tract, therefore oral supplementation should be considered no earlier than 5 days after the last injection. In conclusion, intramuscular or intravenous iron supplementation is carried out only in the hospital, where it is possible to observe the patient and react quickly when intolerance to the given preparation occurs. Iron administered intramuscularly often exposes the patient to taste disturbances (metallic taste in the mouth), which are usually of a transient nature. Less common are general symptoms such as headache, dizziness, shortness of breath, palpitations or gastrointestinal symptoms (abdominal pain, nausea, vomiting, diarrhea). At the injection site, pain, bleeding, abscess, necrosis or local tissue atrophy may occur. Although intravenous iron administration gives satisfactory effects mainly expressed in the increase of hematological parameters and good tolerance, there is still a lack of research and thus no evidence of their safe use in the long term [71].Therefore, oral supplementation in iron deficiency remains the method of choice in the treatment of anemia of chronic diseases, regardless of its severity. The presence of free iron in the blood is dangerous, it can promote the development of infections or exacerbate an ongoing inflammatory process. It can also lead to overloading of this element in the body (secondary hemochromatosis), which may later be manifested by diabetes, liver, heart or kidney failure. However, iron supplementation is contraindicated in patients with active inflammatory process and sepsis [71].

## 12. Folic Acid

The high demand for iron needed for hemoglobin synthesis also requires an adequate supply of folic acid and vitamin B_12_. Folic acid participates in the synthesis of purine and pyrimidine bases forming deoxyribonucleic acid and in the transformation of amino acids and formates. It is necessary for proper cell division, and therefore it plays an important role in tissues characterized by high proliferative intensity, including the hematopoietic system. It is also absolutely essential in the process of producing a myelin sheath of nerve fibers. The daily requirement of folic acid in an adult humans is about 400 µg. Folic acid deficiency is most often manifested by megaloblastic anemia accompanied by leukopenia and thrombocytopenia. Supplemented by women during the preconception period and during pregnancy, it reduces the risk of birth defects associated with abnormal neural tube closure. That is why the demand for folic acid increases significantly during pregnancy and breastfeeding. Its sources are liver, kidneys, yeast, green vegetables and nuts. Treatment of folic acid deficiency consists mainly of its oral supplementation and consumption of the above-mentioned products. Orally administered, it is well absorbed from the small intestine, binds to plasma proteins and goes to the liver, where it is stored and metabolized [60]. It should be remembered that a high supply of folic acid often masks B_12_ deficiency, which can contribute to cognitive decline, especially in the elderly [72].

## 13. Vitamin B_12_

Vitamin B_12_ (cyanocobalamin) is a water-soluble vitamin that regulates hematopoietic processes and the functioning of the nervous system (including myelin synthesis). Like folic acid, it participates in the synthesis of nucleic acids. The average daily requirement for cyanocobalamin is 1–2 µg. Deficiency of this vitamin leads to megaloblastic anemia, changes in peripheral nerves, followed by degenerative changes of the spinal cord in the range of posterior cords and cortical spinal ducts. A deficiency that persists for more than 3 months may result in consolidation of changes in the nervous system. The source of vitamin B_12_ is mainly animal-derived food. Therefore, its supplementation is recommended for people on a vegetarian diet. Released from food under the influence of gastric juice, vitamin B_12_ is combined by an intrinsic factor (IF), which is a glycoprotein produced by the stomach’s parietal cells. The complex formed reaches the small intestine, where cyanocobalamin is disconnected and absorbed in the presence of calcium ions into the portal circulation. The transport of vitamin B_12_ in plasma occurs with the support of binding proteins (transcobalamin I and II) and, as in the case of folic acid, ends in the liver—the organ responsible for the storage of vitamin B_12_.Cyanocobalamin is very poorly absorbed from the gastrointestinal tract after oral administration (about 1%), so at the time of its deep deficiency other forms of supplementation (usually intramuscular) are recommended. Deficiencies in iron or folic acid may reduce or completely inhibit the response to cyanocobalamin treatment. Therefore, the concentration of all three hematopoietic factors should be monitored simultaneously and, if necessary, their supplementation should be implemented [61]. Advantages and disadvantages of various hematopoietic factors supplementation taking account the routes of administration are presented in the Table 2 below.

## 14. Treatment Considerations

The unsatisfactory effect of the above methods in combating anemia of chronic diseases prompts us to continue looking for other possible forms of treatment for this condition. The greatest hopes are currently associated with blocking the action of hepcidin or limiting its hepatic production. It has been observed that heparin administered prophylactically to cancer patients significantly reduces the concentration of this protein in the blood serum. A similar phenomenon was observed among mice [73]. This is due to the binding of bone morphogenetic protein6 (BMP6) to heparin. Bone morphogenetic protein6 is a cytokine from the transforming growth factor (TGF-β) family which, through auto- and paracrine effects, stimulates hepcidin gene transcription in the liver [74]. Reduced hepcidin production has also been observed in patients with rheumatoid arthritis receiving anti-TNF-α antibody (golimumab) [75]. An even more promising result is the improvement of hematological parameters in patients with non-small-cell lung carcinoma who received anti-IL-6 antibody [76]. Also noteworthy is the thiamine derivative fursultiamine, which in vitro function antagonistically to the hepcidin receptor, i.e., ferroportin [77]. The only indication for its use is vitamin B_1_ deficiency. A form of causal treatment of anemia associated with malignant diseases is the use of transferrin conjugates to carry drugs, e.g., cytostatics. Complex of transferrin with doxorubicin—an anthracycline antibiotic commonly used in the treatment of leukemia, lymphoma or sarcoma, allows better penetration of this drug into cancer cells that show much higher expression of transferrin receptors than healthy cells. Such directed action on neoplastic cells allows, among others avoid cardiotoxicity of doxorubicin, which was proved for instance in the studies of Kratz et al. where mice were given doxorubicin conjugate with transferrin at a dose three times higher than the concentration of free drug, while showing a significant reduction of side effects [78,79]. Recently, HIF-2α stabilizers and PHD inhibitors have attracted a lot of interest. Hypoxia induces hypoxia-induced factor (HIF), which stimulates the synthesis of erythropoietin (EPO) and reduces hepcidin production in the liver. Inhibition of the prolyl hydroxylase enzyme (PHD) stabilizes hypoxia-induced factor (HIF), maintaining its positive effect on erythropoiesis and iron metabolism. PHD inhibitors are now a new form of pharmacological treatment of anemia associated with chronic diseases. Many active PHD inhibitors such as roxadustat, molidustat, vadadustat and desidustat are already in a late phase of clinical trials [80].

## 15. Conclusions

1. Anemia of chronic diseases is still a type of anemia that is difficult to treat. This is the result of the complex pathomechanism of this disease and the ability of the underlying disease to make a multifactorial, pathological modulation of the erythropoiesis process.
2. Improper therapeutic management may result from diagnostic errors, inappropriate treatment of the underlying disease and underestimation of the benefits of hematopoietic factors supplementation.
3. Administration of recombinant erythropoietin analogues in patients during chemotherapy allows us to reduce the necessity of transfusion of blood products, although the indications for their use are very limited.
4. The supplementation of hematopoietic factors should be implemented simultaneously with the diagnosis of the underlying disease and last until its cure or longer, with the exception of recombinant erythropoietin analogues and derivatives.
5. Proper nutrition and prevention of food deficiencies remains the primary form of preventing any type of anemia, including anemia of chronic diseases.
6. Better knowledge of proteins and mechanisms involved in the formation of anemia of chronic diseases associated with, among others, malignant neoplasms gives a great chance of creating molecularly targeted drugs for the treatment of these diseases. Potentially the most effective is the inhibition of hepcidin production and activity, therapy with transferrin conjugates with anti-cancer drugs, and increasing iron absorption from the gastrointestinal tract and synthesis of erythropoietin using PHD inhibitors and HIF-2 α stabilizers.

## Figures and Tables

**Table 1:** Comparison of iron deficiency anemia with anemia of chronic disease. (modified based on 14, 31).

Feature	Iron Deficiency Anemia	Anemia of Chronic Disease
**the presence of other chronic diseases**	rarely	often
**average baseline hemoglobinconcentration**	≥9 g/dL	≤9 g/dL
**serum iron concentration**	decreased	significantly decreased
**MCV/MCH**	decreased	normal
**serum ferritin concentration**	low	increased
**serum hepcidin concentration**	low	high
**percentage of reticulocytes in the blood serum**	high	low
**serum folic acid concentration**	normal	decreased
**serum vitamin B_12_ concentration**	normal	decreased
**serum creatinine concentration**	normal	increased
**serum erythropoietin concentration**	increased	decreased

**Table 2:** Summary of the advantages and disadvantages of hematopoietic factors supplementation depending on the route of administration.

Type of Hematopoietic Factor	Route of Administration	Advantages	Disadvantages
**iron**	oral	high safety of use, absence of non-transferrin bound iron (NTBI) in the blood.	limited effectiveness, poor absorption, interaction with other drugs, nausea, vomiting, constipation, diarrhea, itching, rash, erythema
	intramuscular	quick correction of deficiency, less frequent dosing, longer effect, less frequent gastrointestinal ailments, an alternative for swallowing disorders	the need for hospital administration, dysgeusia, headache and dizziness, palpitations, shortness of breath, bleeding, abscess, skin necrosis at the injection site
	intravenous	quick correction of deficiency, less frequent dosing, longer effect, less frequent gastrointestinal ailments, an alternative for swallowing disorders	the need for hospital administration, possible anaphylactic reaction, possible development of infection or exacerbation of sepsis, risk of iron overload, a sharp increase or decrease in blood pressure
**vitamin B_12_**	oral	good tolerability, low risk of overdose, rather as maintenance treatment	poorly absorbed from the gastrointestinal tract (1% of the dose), difficulties in compensating for deficiency
	intramuscular	the method of choice in supplementing the large deficiency, longer effect, less frequent dosing	pain at the injection site, rarely anaphylactic shock and death, and hypersensitivity reactions, pruritus, rash, transient diarrhea
**folic acid**	oral	the method of choice, good tolerance, well absorbed from the gastrointestinal tract, low risk of overdose	allergic skin reactions, gastrointestinal disorders, nausea, vomiting, sleep disturbance, depression or agitation
